# Laparoscopic fundoplication *versus* laparoscopic Roux-en-Y gastric bypass for gastro-oesophageal reflux disease in obese patients: protocol for a randomized clinical trial

**DOI:** 10.1093/bjsopen/zrac132

**Published:** 2022-11-28

**Authors:** Adam Frankel, Iain Thomson, Ayesha Shah, Chen Chen, Syeda Farah Zahir, Andrew Barbour, Gerald Holtmann, B Mark Smithers

**Affiliations:** Princess Alexandra Hospital, Woolloongabba, Queensland, Australia; Faculty of Medicine, The University of Queensland, Herston, Queensland, Australia; Princess Alexandra Hospital, Woolloongabba, Queensland, Australia; Faculty of Medicine, The University of Queensland, Herston, Queensland, Australia; Princess Alexandra Hospital, Woolloongabba, Queensland, Australia; Faculty of Medicine, The University of Queensland, Herston, Queensland, Australia; School of Biomedical Sciences, The University of Queensland, St Lucia, Queensland, Australia; Queensland Facility for Advanced Bioinformatics, Queensland Cyber Infrastructure Foundation, Queensland, Australia; Princess Alexandra Hospital, Woolloongabba, Queensland, Australia; Faculty of Medicine, The University of Queensland, Herston, Queensland, Australia; Princess Alexandra Hospital, Woolloongabba, Queensland, Australia; Faculty of Medicine, The University of Queensland, Herston, Queensland, Australia; Princess Alexandra Hospital, Woolloongabba, Queensland, Australia; Faculty of Medicine, The University of Queensland, Herston, Queensland, Australia

## Abstract

**Background:**

Laparoscopic fundoplication (LF) is the standard surgical procedure for the treatment of gastro-oesophageal reflux disease (GORD). Laparoscopic Roux-en-Y gastric bypass (LRYGB) is commonly performed to achieve weight loss in obese patients, but it also has anti-reflux properties. Hence, in the obese population suffering from GORD, LRYGB could be an alternative to LF. The aim of this trial will be to compare LF and LRYGB in an obese population presenting with GORD and being considered for surgery.

**Methods:**

This will be an investigator-initiated randomized clinical trial. The research population will be obese patients (BMI 30–34.9 with waist circumference more than 88 cm (women) or more than 102 cm (men), or BMI 35–40 with any waist circumference) referred to a public hospital for consideration of anti-reflux surgery. The primary aim of the study will be to determine the efficacy of LF compared with LRYGB on subjective and objective control of GORD. Secondary aims include determining early and late surgical morbidity and the side-effect profile of LF compared with LRYGB and to quantify any non-reflux benefits of LRYGB (including overall quality of life) compared with LF.

**Conclusion:**

This trial will determine whether LRYGB is effective and acceptable as an alternative to LF for the surgical treatment of GORD in obese patients

**Registration number:** Australian New Zealand Clinical Trials Registry (ANZCTR): ACTRN12622000636752p (https://www.anzctr.org.au/).

## Teaser

This is an investigator-initiated randomized clinical trial of laparoscopic fundoplication (LF) *versus* laparoscopic Roux-en-Y gastric bypass (LRYGB) for the treatment of gastro-oesophageal reflux disease (GORD) in obese patients (BMI 30–34.9 with waist circumference more than 88 cm (women) or more than 102 cm (men), or BMI 35–40 with any waist circumference). The primary outcome will be the difference in treatment success (defined as a reduction of 50 per cent of more in the GERD-HRQL score between the preoperative and 1 year values) after LRYGB compared with LF. Secondary outcomes will include identification of any difference in treatment success at 5 years (decrease of 50 per cent of more in the GERD-HRQL score between the preoperative value and the 5-year value), objective measures of GORD at 1 and 5 years (ambulatory pH study and endoscopy), non-GORD gastrointestinal symptom scores at 1 and 5 years, and subjective and objective metrics of overall health at 1 and 5 years.

## Introduction

The Montreal definition of gastro-oesophageal reflux disease (GORD) is ‘a condition, which develops when the reflux of stomach contents causes troublesome symptoms and/or complications’, with ‘troublesome’ being defined as negatively impacting the person’s wellbeing^1^. Recent data from Australian patients who see a general practitioner show that the prevalence of diagnosed GORD is 11.6 per cent^2^. The pathophysiology of GORD is complex, multifactorial, and still only partially understood, as is the perception of GORD symptoms^3^. Widely accepted indications for surgery for GORD include ongoing troublesome symptoms or complications (for example, reflux oesophagitis) despite maximal medical therapy or intolerance of medical therapy^4^. Laparoscopic fundoplication (LF) is the standard surgical procedure for the treatment of GORD.

Obesity is now a pandemic across much of the Western world. Despite well described limitations, BMI (defined as weight in kg divided by height in m^2^) is still the predominant metric used to describe overweight or obese patients^5^. The WHO uses waist circumference in addition to BMI to describe risk classes. ‘Very high risk’ obesity is defined as a BMI 30–34.9 with waist circumference more than 88 cm (women) or more than 102 cm (men), or BMI 35–45 with any waist circumference. Population-based studies of overweight and obesity in Australia show that it is prevalent and the trend is going slowly upward^6^. Data from the 2014–2015 National Health Survey showed that 36 per cent of Australian adults are overweight and 28 per cent are obese^7^, slowly increasing in 2017–2018 to 36 per cent and 31 per cent respectively^8^.

Surgical weight loss is unequivocally superior to non-operative alternatives in terms of excess bodyweight loss, durability of weight loss, improvement in obesity-related co-morbidities, and a reduction in mortality^9,10,11^. It also has ‘non-medical’ benefits, including better physical and mental quality of life and improved social outcomes such as reduced employment impairment and greater productivity^12^. Importantly, a recent meta-analysis showed that bariatric surgery results in an increased median life expectancy for both diabetic and non-diabetic patients (9.3 and 5.1 years respectively^13^). While many types of surgery have been described, laparoscopic Roux-en-Y gastric bypass (LRYGB) seems to achieve the best balance of risks and benefits^14–16^. LRYGB involves the creation of a small gastric pouch, then reconfiguration of the proximal small bowel before joining it on the gastric pouch such that food and digestive juices do not mix for the first 1 to 1.5 metres of the gut. A large multicontinental cohort from academic centres showed a 90-day mortality rate of 0.05 per cent^17^. Indeed, the risks of LRYGB for obesity and related diseases (including GORD) compare favourably with LF performed for GORD in American academic centres, with fewer in-hospital complications and similar mortality, duration of stay and costs^18^. Longer-term data also provide evidence of safety; 546 of 23 450 patients (2 per cent) undergoing LRYGB for obesity died during a follow-up of 216 413 patient-years, less than half that of a matched obese cohort undergoing medical management alone^13^.

Support for surgically induced weight loss in lower BMI groups has been evolving over the last 10 years^19,20,21^. Given that GORD is clearly an obesity-related disease^22,23^, a ‘bariatric’ operation with anti-reflux properties (LRYGB) should clearly be considered as an alternative to LF in obese patients with GORD. The mechanism of GORD control after LRYGB is poorly understood and may be due to the anatomical reconfiguration of LRYGB, the weight loss it induces, or both.

For a patient with a normal BMI and an established diagnosis of GORD who has met the indications for surgery, the standard procedure is LF^24^. For a patient with complicated class 2 or class 3 obesity who also meets the surgical criteria for GORD, the priority is arguably weight loss rather than GORD. From an overall health perspective, the evidence suggests they are better served by bariatric surgery, which brings, among other benefits, an increased life expectancy^11^. Therefore, LRYGB would usually be the procedure of choice because unlike other bariatric procedures, it could achieve the dual benefits of weight loss and improvement in GORD^25,26^.

However, in patients with class 1 or uncomplicated class 2 obesity wanting surgery for GORD, there is clinical equipoise. There is conflicting evidence regarding the outcomes of LF in obese patients^26^. Many cohort series show that when compared with non-obese patients, LF is as safe and effective at improving GORD in obese patients^27–29^. Yet some series have showed reduced control of GORD and greater technical difficulty (measured as longer operating times, more complications, and longer duration of stay)^25,30^. Recurrence of GORD seems to be a particular problem in obese patients in the long term (more than 10 years), with more than 25 per cent having troublesome symptoms and objective evidence of GORD^31,32^. In addition, LF is not generally thought to achieve significant weight loss or improvement in obesity-related complications, which is important to appreciate, because the natural history of patients with class I or uncomplicated class II obesity is likely progression to a higher class^33^.

In contrast, these patients would not meet the current National Institutes of Health criteria for bariatric surgery and would not be eligible for LRYGB within an Australian bariatric surgical programme. Yet LRYGB may be just as effective at treating GORD in this group^34^ and also bring additional potential benefits secondary to weight loss^25^. This makes LRYGB attractive from a public health perspective because there are clear data for bariatric surgery being an effective primary^35^, secondary^36^, and tertiary^37^ preventative health strategy. This explains the results from a recent survey of upper gastrointestinal surgeons, which showed support for LRYGB as the operation of choice for GORD in higher BMI groups and called for national guidelines to be updated to reflect this^38^. Some specialty societies unequivocally recommend LRYGB when BMI is greater than 35 but note that further studies are required to define its role when BMI is 30–35^25,39,40^; however, no randomized clinical trials (RCTs) comparing the standard of LF exist to aid in decision-making. The aim of this randomized trial is to compare LF and LRYGB in an obese (class 1 and 2) population presenting with GORD and being considered for surgery.

## Methods

### Trial design

This was an investigator-initiated parallel group RCT.

### Participants

All patients referred to public outpatient clinics at approved sites for consideration of anti-reflux surgery will be screened. They must have a clinical history of consistent GORD and an ambulatory pH study confirming GORD (defined using the percentage of time with acid reflux). They will have their height and weight recorded and BMI calculated. Those with WHO-defined ‘very high risk’ obesity (BMI 30–34.9 with waist circumference more than 88 cm (women) or more than 102 cm (men), or non-diabetic patients with BMI 35–40 and any waist circumference) will be further screened using the eligibility criteria below. Those meeting the criteria will be offered participation.

Patients will be counselled appropriately on the current state of knowledge, the questions to answer, and the methods proposed to answer them. This will entail a discussion of the two arms of the trial, the pre- and postoperative requirements, and an extensive information-exchange process to achieve national standards of informed consent for the two surgical procedures, which involves discussing both general and material risks (risks specific to that patient’s circumstances). The current standard of care (investigation and treatment pathways) will be outlined if patients decline participation. Consecutive patients who agree to participate will be randomized to undergo LF or LRYGB.

Inclusion criteria will be:

• Aged 18 years or older and 70 years or less
• All sexes
• Troublesome symptoms despite maximal medical therapy with 24-h pH and impedance monitoring supportive of the diagnosis
• BMI 30–40
  • BMI 30–34.9 with waist circumference
    • More than 88 cm (women)
    • More than 102 cm (men)
  • BMI 35–40 with any waist circumference
• Able and willing to give written consent
• Willing to perform the questionnaires, investigations, and postoperative follow-up required for this study
• Able to access regular medical care (in the event of postoperative complications such as internal hernia, and for nutritional screening and supplementation measures)
• Suitable for either surgery

Exclusion criteria will be:

- Intrathoracic stomach (hiatus hernia with more than 50 per cent of stomach in thorax)
- Previous gastro-oesophageal surgery (bariatric, anti-reflux, ulcer, or motility disorder)
- Inflammatory bowel disease
- Multiple surgeries in abdominal cavity or previous small bowel pathology or resection
- Clinical or manometry findings concerning for a severe oesophageal dysmotility syndrome (recognizing that clinically silent manometry-defined motility disorders are common in obesity)
- Any medical condition deemed by an investigator to render the patient ineligible (for example, conditions that may be exacerbated by gastric bypass surgery such as osteoporosis)
- Pregnant or lactating females (routine preoperative pregnancy test for all female patients with child-bearing potential)
- Endocrine cause of obesity
- Current smoker
- Drug or alcohol abuse
- Psychological disorders (for example bulimia or depression)

### Preoperative work-up

All patients will complete symptom and quality-of-life assessments for GORD health-related quality of life (GERD-HRQL)^41^, gastrointestinal health (structured assessment of gastrointestinal symptoms scale; SAGIS)^42^ and overall health (Short-Form Six-Dimension version 2; SF-6Dv2)^43,44^. All patients will undergo global health screening and optimization of medical co-morbidities. There will be a focus on obesity-related conditions (for example, type 2 diabetes mellitus (fasting blood sugar level and glycated haemoglobin (HbA1c)), cardiovascular disease (cardiac risk assessment, 12-lead ECG ± echocardiogram ± exercise stress testing), non-alcoholic fatty liver disease (liver function tests ± abdominal ultrasound), obstructive sleep apnoea (STOP-BANG questionnaire ± sleep physician review),^45^ and nutrition (clinical assessment and protocolized blood tests)), with operating risk scoring conducted with the Elixhauser score^46,47^.

All patients will undergo blood tests to assess nutritional and hormonal parameters, oesophago-gastro-duodenoscopy (OGD) with oesophageal biopsy, 24-h pH and impedance study, solid-state high-resolution oesophageal manometry (reporting using the Chicago IV classification), breath tests (gastric emptying and small intestinal bacterial overgrowth), and bladder pressure measurements. To minimize patient discomfort and achieve the most reliable results, the bladder pressure measurement will be performed at the time of OGD while under sedation. The ambulatory pH probe will be placed at the conclusion of the OGD, again under sedation to minimize discomfort.

In keeping with enhanced recovery after surgery (ERAS) principles^48^, both patient groups will receive operation-specific preoperative counselling about their procedure and recovery interval including expectation setting regarding usual milestones of recovery. All patients will receive counselling and printed literature (pre-existing through Metro South Dietetics service) on how to implement a very-low-calorie diet for 2 weeks before surgery, which can achieve up to 80 per cent reduction in hepatic steatosis^49^.

### Perioperative care

All patients will undergo a general anaesthetic using ERAS principles (goal-directed fluid therapy, aggressive prophylaxis against postoperative nausea and vomiting with routine multimodal anti-emetics, avoidance of volatile agents, opiate minimization, same day resumption of oral intake and mobilization, avoidance of routine drain or nasogastric tube placement). Patient positioning as well as laparoscopic port size and placement will be at the discretion of the operating surgeon. All patients will undergo hiatal dissection and repair based on intraoperative findings but with an aggressive approach, remembering that the indication for surgery is GORD and that hiatus hernia is common and a major contributor to GORD in overweight or obese patients.

### Interventions

Patients will be randomized to either LF or LRYGB, with LF being designated as the ‘control’ procedure. Both operations are widely used in contemporary practice.

For LF, the patient will be supine or placed in lithotomy position. Four laparoscopic ports and an epigastric retractor will be placed. A dissection of the oesophageal hiatus will be performed, preserving both vagus nerves, and a cruroplasty will be performed as required using non-absorbable sutures. Sufficient fundal mobility will be achieved to allow a partial fundoplication (anterior 180 degree or posterior 270 degree) and fixation of the fundus to the diaphragmatic crura and oesophagus will be achieved with non-absorbable sutures. In the absence of evidence showing major differences in long-term GORD symptom control or dysphagia for these two styles of fundoplication, the wrap type as well as division of the superior short gastric vessels and use of a calibrating device will be at the discretion of the operating surgeon^50^. Crural dissection, cruroplasty, and fixation of the distal oesophagus and fundus will be performed as necessary. Operating technique will be recorded to allow possible future analyses.

For LRYGB, the patient will be supine or placed in lithotomy position. Four laparoscopic ports and an epigastric retractor will be placed. A dissection of the oesophageal hiatus will be performed, preserving both vagus nerves, and a cruroplasty will be performed as required using non-absorbable sutures. Dissection at the mid-lesser curve and at the cardia will allow an assessment of the lesser sac, with successive stapler firings to create a small gastric pouch. The gastric pouch will be calibrated using a 34-Fr bougie and a target length of 7 cm. The gastrocolic omentum may be split vertically at the surgeon’s discretion to allow safe delivery of the small bowel. The jejunum will be divided to give a biliopancreatic limb length of approximately 70 cm^51,52^. The alimentary limb will be measured to a length of 70–120 cm (tailored by the surgeon to the patient’s BMI)^53^. With regard to this study’s outcome measures, there is a lack of evidence to support hand-sewn, circular, or stapled anastomoses for either the gastro-jejunostomy or the jejuno-jejunostomy. Hence, again recognizing individual surgeon preferences and competence, the specific anastomotic techniques used will be at the operating surgeon’s discretion but will be recorded for possible future analyses. The mesenteric defect and Peterson’s space will be closed with non-absorbable sutures^54^.

### Postoperative care

Postoperative recovery and discharge will be in keeping with standard clinical practice. This includes multimodal analgesia with intra- and postoperative opiate minimization, early mobilization with physiotherapy input, graduated compression stockings, and low molecular weight heparin using local evidence-based guidelines, immediate cessation of anti-acid medication^55^, and no routine postoperative imaging. The target in-hospital stay is 1 night^56^. Once recovered from the anaesthetic, all patients will commence a clear liquid diet (low calorie, low sugar, non-carbonated, caffeine-free, and alcohol-free)^57^. Progression to full liquids (high protein, low calorie, and low sugar) will occur on day one after surgery with slow introduction of pureed or mashed food over the following weeks at the discretion of the operating surgeon.

At 1, 3, 6, 12, 24, 36, 48, and 60 months after surgery, all patients will repeat the quality-of-life assessments, be weighed, and have blood tests to assess nutritional and hormonal parameters. At 1 and 5 years, all patients will undergo repeat global health assessment, OGD with oesophageal biopsy, 24-h pH + MII study, oesophageal manometry, breath tests (gastric emptying and small intestinal bacterial overgrowth), and bladder pressure measurements. In keeping with published Australian recommendations^58^, and guided by some of the blood test results from above, LRYGB patients will receive ongoing long-term dietitian review and nutritional supplementation.

### Objectives

The primary objective is to determine if there is a difference in treatment success (defined as a reduction of 50 per cent of more in the GERD-HRQL score between the preoperative value and the 1-year value) following LRYGB compared with LF (as used in two contemporary high-impact GORD treatment RCTs^59,60^).

With this respect, the trial was design according to the following hypotheses:

*H_0_*: Postoperative GORD symptom scores (GERD-HRQL) will be similar for patients undergoing LRYGB *versus* LF for GORD at 1 year.
*H_A_*: Postoperative GORD symptom scores (GERD-HRQL) will be different for patients undergoing LRYGB *versus* LF at 1 year.

The secondary objectives are to determine any differences in terms of:

- Treatment success at 5 years (decrease of 50 per cent of more in the GERD-HRQL score between the preoperative value and the 5-year value)
- Objective measures of GORD at 1 and 5 years
- Non-GORD gastrointestinal symptom scores at 1 and 5 years
- Overall health at 1 and 5 years

### Outcomes

While expert opinion and cohort studies are readily available, the question of which of two commonly performed operations provide the best treatment for GORD in the obese population will not be settled until a RCT is performed. GORD is one of the most common medical conditions afflicting the Western population, and rates of obesity continue to increase and will remain a problem for generations to come. As such, the number of patients to whom the trial findings can be applied in the future are very large. Analysis of local data confirms that the number of patients seeking surgical treatment for GORD in our health district is slowly increasing, as is their BMI. In addition, fundamental questions regarding oesophagogastric pathophysiology in obese patients with GORD are unanswered currently. This is in large part due to a piecemeal approach in the literature to date. Similarly, there has never been a randomized trial of two surgical procedures of similar magnitude where only one is expected to induce weight loss; this brings a unique opportunity to study gut neurohormonal changes after a bypass operation. Insights gained by the formal, in-depth study of pre- and postoperative physiological parameters will greatly add to the basic understanding of this patient population and undoubtedly lead to further advances in the field.

For participants in both control and treatment groups, outcomes will be measured at baseline, 1 year and 5 years after surgery. The primary outcome is GORD symptom scores (assessed with the GERD-HRQL survey). Secondary outcome(s) are GORD symptom scores (assessed with the GERD-HRQL survey) at 5 years, the percentage of total time spent with pH < 4 in the oesophagus, the presence and severity of oesophagitis, and patient-reported outcomes. Gastrointestinal-specific quality of life will be assessed using the SAGIS questionnaire^42^ (including dysphagia, belching, bloating, flatulence, and altered bowel habit), while overall quality of life will be assessed using SF-6Dv2^43,44^. The operative metrics and surgical morbidity measures will be operating time (measured in minutes), pain score (measured using a visual analogue score and oral morphine equivalents), length of hospital stay (measured in days), the Comprehensive Complication Index^61^, the development of complicated gallstone disease, and readmission rate (at 90 days). Bodyweight changes will be monitored. With respect to the changes in pathophysiological parameters contributing to GORD, the transient lower oesophageal sphincter relaxation episodes and duration on high-resolution manometry and bladder pressure measurement (as a surrogate for intra-abdominal pressure) will be measured both before and after surgery.

For randomization, an adaptive procedure will be used to stratify patients according to sex, BMI (class I or uncomplicated class II), and percentage of time with oesophageal pH < 4 (4–10 per cent and more than 10 per cent), with the use of a ‘biased coin’ procedure to balance treatment assignments^60^. The symmetric Kullback–Leibler divergence minimization method, which allows the use of both continuous and categorical factors will be used^62^. A 1:1 assignment will be used with a block size of six. The goal is that the patient will be kept blinded until after the operation. To achieve this, group allocation will be performed by a database manager (non-clinician) the morning before surgery (to facilitate operating room scheduling and equipment preparation). It will be directly communicated to the operating surgeon.

For blinding, the allocation sequence will be concealed from researchers responsible for assigning participants. The patient will be kept blinded until after the operation. Where possible, data analysis will be conducted in a blinded fashion.

### Statistical considerations

There are limited data to guide the sample size calculation. A pilot phase of 20 patients (10 in each arm)^63^ will be completed at the Upper Gastrointestinal Surgery Unit, Princess Alexandra Hospital. This will allow an assessment of recruitment rate and an analysis of the primary outcome to enable the power calculations and trial duration to be recalculated and ensure that the trial remains practical and can be completed. In addition, any initial logistical issues can be resolved. Thereafter, the trial will be extended across Logan Hospital, QEII Jubilee Hospital and the Royal Brisbane and Women’s Hospital.

The trial will be powered based on the primary outcome alone. Using *α* = 0.05, *β* = 0.8, a randomization ratio of 1: 1 and a mean(s.d.) GERD-HRQL score of 8(8) after LF in class I and II obesity^64^ and 5 after LRYGB^65^, a sample size of 164 would be required. Assuming a 10 per cent dropout, there would need to be 91 patients in each arm. This equates to approximately 2 years of referrals of eligible patients to the Princess Alexandra Hospital alone. Assuming participation of the other hospitals and 50 per cent uptake, a conservative estimate is that recruitment will take approximately 3 years.

Acknowledging the lack of data to guide power calculations and hence the possibility that the alternative treatment (LRYGB) may give the same mean GERD-HRQL score as the control treatment (LF), a planned non-inferiority analysis will also be performed. Using *α* = 0.05, *β* = 0.9, a randomization ratio of 1: 1, a mean(s.d.) GERD-HRQL score of 8(8) after LF in class I and II obesity and a non-inferiority limit (d) of 4, a sample size of 138 is required. Assuming a 10 per cent dropout, there would need to be 77 patients in each arm.

Given that both superiority and non-inferiority analyses are planned, it is appropriate to perform both intention-to-treat and per-protocol analyses. Continuous data will be tested using a Student’s *t* test if data are normally distributed or otherwise a Mann–Whitney *U* test. Normality of continuous data will be assessed with the Shapiro–Wilks test. Categorical data will generally be assessed using the chi-squared test or Fisher’s exact test when the assumptions of the chi-squared test are not met. All *P* values will be two-tailed (except for non-inferiority analysis), and significance will be set at 0.050.

### Ethical considerations

The research project will be conducted in full conformance with principles of the Declaration of Helsinki, Good Clinical Practice and within the laws and regulations of Queensland and Australia. All Metro South Health Research requirements will be complied with at all times.

All participating surgeons are Australian-trained and Fellows of the Royal Australasian College of Surgeons. All have had post-vocational fellowship placements in the sub-specialty of upper gastrointestinal surgery. All have public hospital appointments as consultant surgeons and have had sufficient training to be granted credentialling in both operation types. All participating physicians are Fellows of the Royal Australasian College of Physicians and are recognized sub-specialists in the field of gastroenterology. All listed investigators have been consulted about the trial and, having had the opportunity to review and optimize the trial protocol, have reached a consensus.

As would occur in standard clinical practice, the recruiting surgeon will be responsible for the provision of sufficient information for the patient to make an informed decision about undergoing an operation. Initially, the surgeon will counsel the patient on what the usual clinical pathway and operation would be (LF) if they decline trial participation, including the benefits of LF as well as general and material risks. The surgeon will also outline the uncertainty about whether the current standard of care (LF) is the best operation in their circumstances. The surgeon will then outline the requirements for trial participation and discuss the alternative operation (LRYGB), including the benefits and risks in sufficient depth for the patient to make an informed decision about participation. Information will be given verbally and in written form. If English proficiency is insufficient, a credentialled translator will be provided, in keeping with usual clinical standards for informed consent. Generally, this process would happen in the outpatient clinic, the patient would return home for an interval of approximately 6 weeks, then return to a second appointment for further discussion and counselling, at which time a decision may be reached. If participation is declined, the patient will revert to the current standard clinical pathway. Consent can be withdrawn at any time, although for obvious reasons this should generally be withdrawn before surgery.

### Operative complications

All patients having a postoperative complication (defined by the Clavien–Dindo and Comprehensive Complication Index) will have their complication recorded and case reviewed. A planned interim analysis at the conclusion of the pilot phase (after 10 cases) will be conducted. The research team will maintain full compliance with the Metro South Hospital and Health Service Research Management Monitoring Procedure, including self-assessment, external review, and annual reports.

### Data management and monitoring

Data will be managed and stored in line with applicable local and national guidelines (Metro South Health Research Management Compliance Framework, National Statement on Ethical Conduct in Human Research (2018), Australian Code for the Responsible Conduct of Research 2018). Data extraction will be performed by the co-ordinating principal investigator. Storage will be online in a secure database (REDCap). Once extracted for analysis, data will be kept on a Queensland Health network drive subject to Enterprise-Level security and within a password-protected file. Being a trial of two surgical interventions that are currently used in daily clinical practice, all participating surgeons will be fully capable of assessing, counselling, and operating on eligible patients. All aspects will be performed in accordance with accepted clinical practice.

It is expected that with any surgery, adverse events (AEs) will be encountered. All AEs will be identified and noted by the responsible clinical team in real time and addressed appropriately in accordance with good clinical practice. All AEs will be captured and recorded. The same holds for serious AEs (SAEs), but these will also be expeditiously reported to the co-ordinating principal investigator, the hospital Morbidity and Mortality Committee and Metro South Research.

A data safety monitoring board will be constituted in keeping with the National Health and Medical Research Council Data Safety Monitoring Boards 2018 guideline. There will be at least three members, of whom one will be a statistician, one will be familiar with GORD, and one will be familiar with clinical trial conduct. None of the members will be involved in any other way with the trial or will have any perceived conflict of interest.

## Discussion

In the Western world, obesity is prevalent and has become an everyday consideration for all surgical specialties. Despite the seemingly obvious mechanical effect of central adiposity driving a refluxogenic pressure gradient from the abdomen towards the thoracic and cervical oesophagus, data to prove the pathophysiological mechanisms of obesity that contribute to GORD are sparse.

From a clinical perspective, a patient presenting with GORD and meeting the indications for surgery could be conceptually placed in one of three distinct groups: normal BMI, elevated BMI not meeting National Institutes of Health (NIH) criteria for bariatric surgery (overweight, class I, or uncomplicated class II obesity), and elevated BMI meeting NIH criteria for bariatric surgery (complicated class II or any class III obesity).

The first group has a well established treatment pathway and the standard procedure is LF. The surgical treatment of GORD in the latter two groups is more controversial. Patients in group 3, despite presenting with GORD, should be considered for bariatric surgery using the 1991 NIH guidelines. Of course, this would necessitate an in-depth discussion about their goals of care and the relative merits of LF and the bariatric surgical options. The limited data available suggest that LF would be safe and effective but it would solely address their GORD and it could be argued that there has been a missed opportunity for more holistic healthcare to be provided. LRYGB is probably the preferred bariatric procedure in this context, both for reliability of weight loss and the improvement of GORD. Laparoscopic adjustable gastric banding and laparoscopic sleeve gastrectomy will both achieve weight loss but are both refluxogenic^30,66^. More recently, single anastomosis gastric bypass (SAGB) has gained popularity for its relative simplicity, similar weight loss, and possibly improved diabetic remission rates compared with LRYGB; however, a recent multicentre RCT comparing the two demonstrated that LRYGB resulted in less GORD, oesophagitis, and gastritis^67^. Notably, dumping syndrome was more frequent after LRYGB. While data on *de novo* reflux symptoms after SAGB are available in the bariatric literature, the population under study was not patients presenting with GORD and hence no inference can be made regarding potential efficacy compared with LF. In contrast, the anatomical alterations of LRYGB that result in weight loss and improvement in obesity-related co-morbidities also prevent both acid and bile reflux. These patients can undergo LF and expect good results, but LRYGB may be just as effective at treating GORD with additional potential benefits secondary to weight loss. This trial aims to add evidence and could help in clarifying which of these procedures may be preferred in such a population, both from the patient’s perspective and using objective metrics, and shed some light on the pathophysiological interplay between obesity and GORD to improve understanding and drive future innovation.

## Funding

A.F. is supported by a joint grant from the Royal Australasian College of Surgeons (Herbert and Gloria Keys Research Scholarship 2022) and the University of Queensland.

## Data Availability

Data sharing is not applicable to this article as no data sets were generated or analysed.
